# 4-[(Adamantan-1-yl)carbamo­yl]pyridinium chloride

**DOI:** 10.1107/S1600536812022866

**Published:** 2012-05-26

**Authors:** Ying-Chun Wang

**Affiliations:** a College of Chemistry and Chemical Engineering, Southeast University, Nanjing 210096, People’s Republic of China

## Abstract

In the title salt, C_16_H_21_N_2_O^+^·Cl^−^, the amide group makes a dihedral angle of 24.98 (2)° with respect to the pyridinium ring. In the crystal, both the amide and pyridinium N atoms are involved in N—H⋯Cl hydrogen bonding. Weak inter­molecular C—H⋯Cl and C—H⋯O inter­actions also occur.

## Related literature

For the structures and properties of related compounds, see: Fu *et al.* (2011*a* ▶,*b* ▶,*c* ▶); Dai & Chen (2011 ▶); Xu *et al.* (2011 ▶); Zheng (2011 ▶).
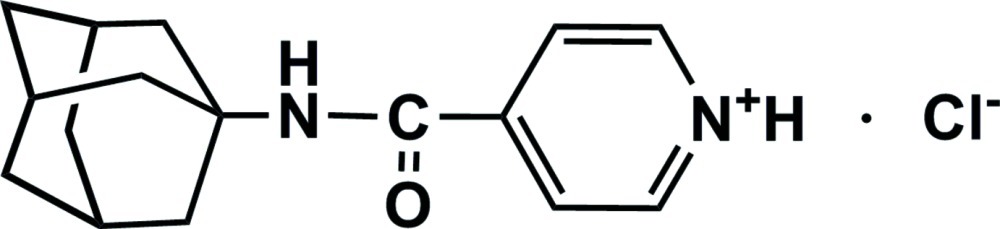


## Experimental

### 

#### Crystal data

C_16_H_21_N_2_O^+^·Cl^−^
*M* *_r_* = 292.80
Monoclinic, 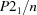

*a* = 7.072 (4) Å
*b* = 22.691 (13) Å
*c* = 8.905 (6) Å
β = 91.703 (7)°
*V* = 1428.3 (15) Å^3^
*Z* = 4
Mo *K*α radiation
μ = 0.27 mm^−1^
*T* = 93 K
0.10 × 0.03 × 0.03 mm

#### Data collection

Rigaku Mercury2 diffractometer
Absorption correction: multi-scan (*CrystalClear*; Rigaku, 2005 ▶) *T*_min_ = 0.910, *T*_max_ = 1.000
13912 measured reflections
3278 independent reflections
2863 reflections with *I* > 2σ(*I*)
*R*_int_ = 0.041

#### Refinement

*R*[*F* ^2^ > 2σ(*F* ^2^)] = 0.044
*wR*(*F* ^2^) = 0.157
*S* = 1.19
3278 reflections
181 parameters
H-atom parameters constrained
Δρ_max_ = 0.54 e Å^−3^
Δρ_min_ = −0.43 e Å^−3^

### 

Data collection: *CrystalClear* (Rigaku, 2005 ▶); cell refinement: *CrystalClear*; data reduction: *CrystalClear*; program(s) used to solve structure: *SHELXS97* (Sheldrick, 2008 ▶); program(s) used to refine structure: *SHELXL97* (Sheldrick, 2008 ▶); molecular graphics: *SHELXTL* (Sheldrick, 2008 ▶); software used to prepare material for publication: *SHELXTL*.

## Supplementary Material

Crystal structure: contains datablock(s) I, global. DOI: 10.1107/S1600536812022866/xu5543sup1.cif

Structure factors: contains datablock(s) I. DOI: 10.1107/S1600536812022866/xu5543Isup2.hkl

Supplementary material file. DOI: 10.1107/S1600536812022866/xu5543Isup3.cml

Additional supplementary materials:  crystallographic information; 3D view; checkCIF report

## Figures and Tables

**Table 1:** Hydrogen-bond geometry (Å, °)

*D*—H⋯*A*	*D*—H	H⋯*A*	*D*⋯*A*	*D*—H⋯*A*
N1—H1*A*⋯Cl1^i^	0.86	2.19	3.020 (2)	161
N2—H2*A*⋯Cl1	0.86	2.51	3.272 (2)	148
C1—H1*B*⋯Cl1^ii^	0.95	2.77	3.520 (3)	136
C2—H2*B*⋯Cl1^iii^	0.95	2.76	3.494 (3)	134
C5—H5*A*⋯O1^iv^	0.95	2.31	3.149 (3)	147

Symmetry codes: (i) 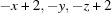; (ii) 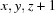; (iii) 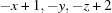; (iv) 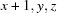.

**Fig. 1.**
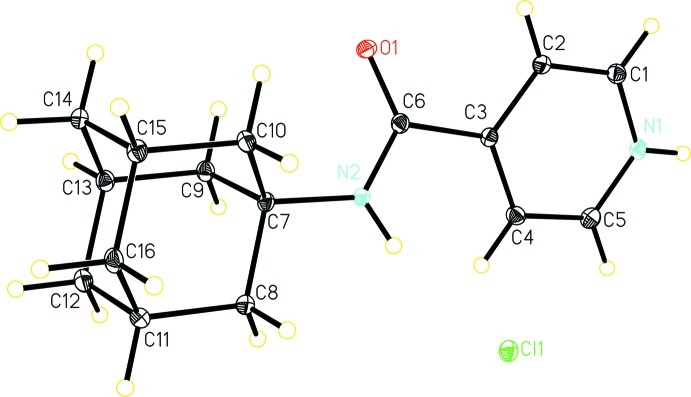
Molecular view of the title compound with the atomic numbering scheme. Displacement ellipsoids are drawn at the 30% probability level.

**Fig. 2.**
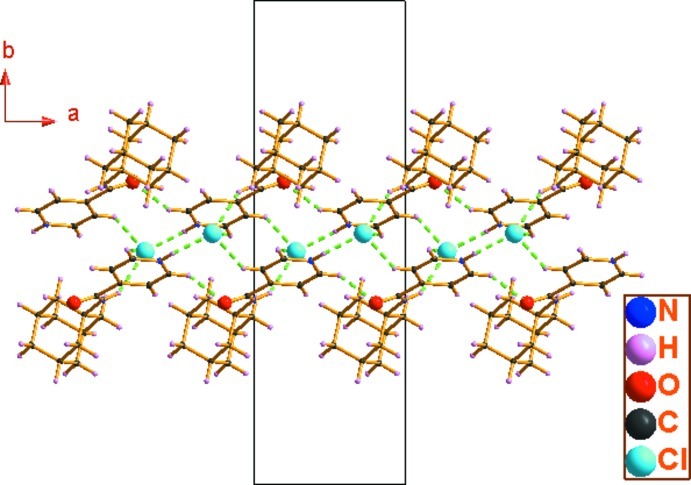
The crystal packing of the title compound viewed along the c axis showing the hydrogen bondings unit (dashed line).

## supplementary crystallographic information

### Comment

Organic amino compounds attracted more attention as phase transition dielectric materials for its application in memory storage (Fu *et al.*, 2011*a*, *b* and *c*). With the purpose of obtaining phase transition crystals of amino compounds, various amines have been studied and we have elaborated a series of new materials with this organic molecules (Dai & Chen 2011; Xu, *et al.* 2011; Zheng 2011). In this study, we describe the crystal structure of the title compound, 4-[(adamantyl)carbamoyl]pyridinium chloride.

The asymmetric unit is composed of one 4-[(adamantyl)carbamoyl]pyridinium cation and one chloride anion. The pyridine N atom was protonated, and the amide group makes a dihedral angle of 24.98 (2)° with respect to the pyridinium ring (Fig.1). The torsion angles of C2—C3—C6—O1 and C2—C3—C6—N2 are 23.6 (3)° and -158.1 (4)°, C4—C3—C6—O1 and C4—C3—C6—N2 are -152.3 (2)° and 26.0 (3)°. Intermolecular N—H···Cl bonds and weak C—H···Cl and C—H···O contacts link ions into a network parallel to (0 1 0) plane (Table 1 and Fig 2).

### Experimental

Isonicotinic acid 5 g was added in thionyl chloride (50 ml), and the mixture reacted at 353 K for 5 h. Then the solvate was removed under reduced pressure, the isonicotinoyl chloride was obtained. The 1-aminodiamantane hydrochloride (10 mmol) and triethylamine 2.02 g (20 mmol) dissolved in chloroform (40 ml) at 273 K, then the isonicotinoyl chloride 1.51 g (10 mmol) was added. Then the reactant mixture was stired for 7 h at room temperature and some flaxen solid appeared. After filtering the mixture, the solid was dissolved in water and was neutralized with sodium carbonate, The mixed solution was extracted by dichloromethane. The N-(1-adamantyl)isonicotinamide was obtained when the dichloromethane was evaporated under reduced pressure. A mixture of N(1-adamantyl)isonicotinamide 2.56 g (10 mmol), HCl (2.0 mL, 6 mol/L), and 20 mL ethanol were added into a 50ml flask and refluxed for 5 hours, then cooled and filtrated. The solution was evaporated slowly in the air. Colorless block crystals suitable for X-ray analysis were obtained after one week.

### Refinement

All H atoms attached to C atoms were fixed geometrically and treated as riding with C-H = 0.95 Å (aromatic), C-H = 0.99 Å (methylene) and C-H = 1.00 Å (methine) with *U*_iso_(H) = 1.2*U*_eq_(C). H atoms bonded to N atoms were located in difference Fourier maps and restrained with the H—N = 0.86 (2)Å. In the last stage of refinement they were treated as riding on the N atoms with *U*_iso_(H) = 1.2*U*_eq_(N).

### Crystal data

**Table:**

C_16_H_21_N_2_O^+^·Cl^−^	*F*(000) = 624
*M**_r_* = 292.80	*D*_x_ = 1.362 Mg m^−^^3^
Monoclinic, *P*2_1_/*n*	Mo *K*α radiation, λ = 0.71073 Å
Hall symbol: -P 2yn	Cell parameters from 3278 reflections
*a* = 7.072 (4) Å	θ = 1.8–27.5°
*b* = 22.691 (13) Å	µ = 0.27 mm^−^^1^
*c* = 8.905 (6) Å	*T* = 93 K
β = 91.703 (7)°	Block, colourless
*V* = 1428.3 (15) Å^3^	0.10 × 0.03 × 0.03 mm
*Z* = 4	

### Data collection

**Table:**

Rigaku Mercury2 diffractometer	3278 independent reflections
Radiation source: fine-focus sealed tube	2863 reflections with *I* > 2σ(*I*)
Graphite monochromator	*R*_int_ = 0.041
Detector resolution: 13.6612 pixels mm^-1^	θ_max_ = 27.5°, θ_min_ = 1.8°
CCD profile fitting scans	*h* = −9→9
Absorption correction: multi-scan (*CrystalClear*; Rigaku, 2005)	*k* = −29→29
*T*_min_ = 0.910, *T*_max_ = 1.000	*l* = −11→11
13912 measured reflections	

### Refinement

**Table:**

Refinement on *F*^2^	Primary atom site location: structure-invariant direct methods
Least-squares matrix: full	Secondary atom site location: difference Fourier map
*R*[*F*^2^ > 2σ(*F*^2^)] = 0.044	Hydrogen site location: inferred from neighbouring sites
*wR*(*F*^2^) = 0.157	H-atom parameters constrained
*S* = 1.19	*w* = 1/[σ^2^(*F*_o_^2^) + (0.0868*P*)^2^ + 0.3419*P*] where *P* = (*F*_o_^2^ + 2*F*_c_^2^)/3
3278 reflections	(Δ/σ)_max_ < 0.001
181 parameters	Δρ_max_ = 0.54 e Å^−^^3^
0 restraints	Δρ_min_ = −0.43 e Å^−^^3^

### Special details

**Table:**

Geometry. All esds (except the esd in the dihedral angle between two l.s. planes) are estimated using the full covariance matrix. The cell esds are taken into account individually in the estimation of esds in distances, angles and torsion angles; correlations between esds in cell parameters are only used when they are defined by crystal symmetry. An approximate (isotropic) treatment of cell esds is used for estimating esds involving l.s. planes.
Refinement. Refinement of F^2^ against ALL reflections. The weighted R-factor wR and goodness of fit S are based on F^2^, conventional R-factors R are based on F, with F set to zero for negative F^2^. The threshold expression of F^2^ > 2sigma(F^2^) is used only for calculating R-factors(gt) etc. and is not relevant to the choice of reflections for refinement. R-factors based on F^2^ are statistically about twice as large as those based on F, and R- factors based on ALL data will be even larger.

### Fractional atomic coordinates and isotropic or equivalent isotropic displacement parameters (Å^2^)

**Table:**

	*x*	*y*	*z*	*U*_iso_*/*U*_eq_	
N2	0.4961 (2)	0.11853 (7)	0.77561 (19)	0.0136 (4)	
H2A	0.6014	0.1044	0.7457	0.016*	
O1	0.3053 (2)	0.12500 (7)	0.97887 (17)	0.0195 (4)	
N1	0.8767 (2)	0.03797 (7)	1.21603 (19)	0.0150 (4)	
H1A	0.9604	0.0230	1.2768	0.018*	
C4	0.8026 (3)	0.08813 (9)	0.9896 (2)	0.0157 (4)	
H4A	0.8425	0.1069	0.9006	0.019*	
C8	0.4853 (3)	0.15358 (9)	0.5188 (2)	0.0154 (4)	
H8A	0.5154	0.1135	0.4830	0.018*	
H8B	0.6058	0.1748	0.5389	0.018*	
C12	0.3246 (3)	0.24905 (9)	0.4539 (2)	0.0169 (4)	
H12A	0.4446	0.2706	0.4735	0.020*	
H12B	0.2500	0.2709	0.3762	0.020*	
C6	0.4548 (3)	0.11078 (9)	0.9207 (2)	0.0143 (4)	
C7	0.3740 (3)	0.14958 (8)	0.6638 (2)	0.0123 (4)	
C3	0.6104 (3)	0.08371 (8)	1.0195 (2)	0.0127 (4)	
C2	0.5567 (3)	0.05623 (9)	1.1516 (2)	0.0153 (4)	
H2B	0.4267	0.0531	1.1743	0.018*	
C9	0.3315 (3)	0.21225 (8)	0.7186 (2)	0.0146 (4)	
H9A	0.2611	0.2103	0.8129	0.018*	
H9B	0.4515	0.2336	0.7395	0.018*	
C13	0.2134 (3)	0.24535 (8)	0.5982 (2)	0.0151 (4)	
H13A	0.1859	0.2861	0.6346	0.018*	
C5	0.9336 (3)	0.06493 (9)	1.0909 (2)	0.0169 (4)	
H5A	1.0648	0.0680	1.0722	0.020*	
C10	0.1881 (3)	0.11626 (8)	0.6305 (2)	0.0143 (4)	
H10A	0.1160	0.1126	0.7237	0.017*	
H10B	0.2160	0.0761	0.5938	0.017*	
C16	0.1811 (3)	0.15352 (9)	0.3664 (2)	0.0161 (4)	
H16A	0.2081	0.1133	0.3296	0.019*	
H16B	0.1052	0.1744	0.2877	0.019*	
C15	0.0694 (3)	0.14989 (9)	0.5109 (2)	0.0146 (4)	
H15A	−0.0520	0.1284	0.4902	0.017*	
C1	0.6932 (3)	0.03367 (9)	1.2493 (2)	0.0165 (4)	
H1B	0.6576	0.0151	1.3398	0.020*	
C14	0.0263 (3)	0.21217 (9)	0.5695 (2)	0.0157 (4)	
H14A	−0.0534	0.2337	0.4946	0.019*	
H14B	−0.0438	0.2095	0.6639	0.019*	
C11	0.3670 (3)	0.18655 (9)	0.3972 (2)	0.0163 (4)	
H11A	0.4396	0.1890	0.3028	0.020*	
Cl1	0.77720 (7)	0.01868 (2)	0.63897 (5)	0.01459 (17)	

### Atomic displacement parameters (Å^2^)

**Table:**

	*U*^11^	*U*^22^	*U*^33^	*U*^12^	*U*^13^	*U*^23^
N2	0.0114 (8)	0.0184 (8)	0.0110 (8)	0.0036 (6)	0.0002 (7)	0.0022 (6)
O1	0.0137 (8)	0.0295 (8)	0.0154 (8)	0.0054 (6)	0.0025 (6)	0.0056 (6)
N1	0.0154 (9)	0.0175 (8)	0.0120 (8)	0.0031 (6)	−0.0045 (7)	0.0002 (6)
C4	0.0164 (11)	0.0172 (10)	0.0135 (10)	−0.0002 (7)	0.0005 (8)	0.0011 (7)
C8	0.0145 (10)	0.0183 (10)	0.0134 (10)	0.0036 (7)	0.0008 (8)	0.0020 (7)
C12	0.0178 (11)	0.0159 (10)	0.0167 (10)	0.0001 (7)	−0.0017 (8)	0.0032 (7)
C6	0.0126 (10)	0.0156 (10)	0.0146 (10)	0.0001 (7)	−0.0017 (8)	0.0015 (7)
C7	0.0109 (9)	0.0141 (9)	0.0117 (9)	0.0010 (7)	−0.0020 (7)	0.0007 (7)
C3	0.0134 (10)	0.0133 (9)	0.0114 (9)	0.0009 (7)	−0.0013 (8)	−0.0009 (7)
C2	0.0137 (10)	0.0173 (9)	0.0149 (10)	0.0018 (7)	0.0004 (8)	0.0019 (8)
C9	0.0161 (10)	0.0145 (10)	0.0130 (10)	0.0011 (7)	−0.0015 (8)	−0.0002 (7)
C13	0.0175 (10)	0.0119 (9)	0.0158 (10)	0.0018 (7)	0.0000 (8)	0.0001 (7)
C5	0.0136 (10)	0.0183 (10)	0.0186 (11)	−0.0007 (7)	−0.0024 (8)	−0.0018 (7)
C10	0.0150 (10)	0.0138 (9)	0.0141 (10)	−0.0011 (7)	−0.0015 (8)	0.0030 (7)
C16	0.0179 (10)	0.0177 (10)	0.0124 (10)	0.0029 (8)	−0.0036 (8)	0.0008 (7)
C15	0.0129 (10)	0.0163 (10)	0.0142 (10)	−0.0014 (7)	−0.0034 (8)	0.0000 (7)
C1	0.0196 (11)	0.0163 (10)	0.0138 (10)	0.0020 (8)	0.0014 (8)	0.0003 (8)
C14	0.0140 (10)	0.0183 (10)	0.0148 (10)	0.0040 (7)	0.0004 (8)	0.0038 (7)
C11	0.0163 (10)	0.0193 (10)	0.0133 (10)	0.0023 (8)	0.0014 (8)	0.0027 (7)
Cl1	0.0136 (3)	0.0161 (3)	0.0139 (3)	0.00244 (16)	−0.00125 (19)	0.00123 (16)

### Geometric parameters (Å, º)

**Table:**

N2—C6	1.345 (3)	C2—C1	1.378 (3)
N2—C7	1.478 (2)	C2—H2B	0.9500
N2—H2A	0.8600	C9—C13	1.536 (3)
O1—C6	1.234 (3)	C9—H9A	0.9900
N1—C1	1.343 (3)	C9—H9B	0.9900
N1—C5	1.343 (3)	C13—C14	1.537 (3)
N1—H1A	0.8600	C13—H13A	1.0000
C4—C5	1.378 (3)	C5—H5A	0.9500
C4—C3	1.396 (3)	C10—C15	1.539 (3)
C4—H4A	0.9500	C10—H10A	0.9900
C8—C7	1.534 (3)	C10—H10B	0.9900
C8—C11	1.542 (3)	C16—C11	1.531 (3)
C8—H8A	0.9900	C16—C15	1.532 (3)
C8—H8B	0.9900	C16—H16A	0.9900
C12—C13	1.529 (3)	C16—H16B	0.9900
C12—C11	1.538 (3)	C15—C14	1.540 (3)
C12—H12A	0.9900	C15—H15A	1.0000
C12—H12B	0.9900	C1—H1B	0.9500
C6—C3	1.518 (3)	C14—H14A	0.9900
C7—C9	1.536 (3)	C14—H14B	0.9900
C7—C10	1.538 (3)	C11—H11A	1.0000
C3—C2	1.394 (3)		
			
C6—N2—C7	124.78 (17)	C12—C13—C9	109.36 (17)
C6—N2—H2A	117.6	C12—C13—C14	110.43 (17)
C7—N2—H2A	117.6	C9—C13—C14	108.90 (16)
C1—N1—C5	122.08 (18)	C12—C13—H13A	109.4
C1—N1—H1A	118.9	C9—C13—H13A	109.4
C5—N1—H1A	119.0	C14—C13—H13A	109.4
C5—C4—C3	119.15 (19)	N1—C5—C4	120.3 (2)
C5—C4—H4A	120.4	N1—C5—H5A	119.8
C3—C4—H4A	120.4	C4—C5—H5A	119.8
C7—C8—C11	109.77 (17)	C7—C10—C15	109.57 (16)
C7—C8—H8A	109.7	C7—C10—H10A	109.8
C11—C8—H8A	109.7	C15—C10—H10A	109.8
C7—C8—H8B	109.7	C7—C10—H10B	109.8
C11—C8—H8B	109.7	C15—C10—H10B	109.8
H8A—C8—H8B	108.2	H10A—C10—H10B	108.2
C13—C12—C11	109.58 (16)	C11—C16—C15	109.68 (16)
C13—C12—H12A	109.8	C11—C16—H16A	109.7
C11—C12—H12A	109.8	C15—C16—H16A	109.7
C13—C12—H12B	109.8	C11—C16—H16B	109.7
C11—C12—H12B	109.8	C15—C16—H16B	109.7
H12A—C12—H12B	108.2	H16A—C16—H16B	108.2
O1—C6—N2	125.65 (19)	C16—C15—C10	108.92 (17)
O1—C6—C3	118.52 (18)	C16—C15—C14	110.33 (16)
N2—C6—C3	115.80 (18)	C10—C15—C14	109.25 (16)
N2—C7—C8	106.92 (16)	C16—C15—H15A	109.4
N2—C7—C9	110.06 (16)	C10—C15—H15A	109.4
C8—C7—C9	108.83 (16)	C14—C15—H15A	109.4
N2—C7—C10	112.01 (16)	N1—C1—C2	119.81 (19)
C8—C7—C10	108.93 (17)	N1—C1—H1B	120.1
C9—C7—C10	110.00 (16)	C2—C1—H1B	120.1
C2—C3—C4	118.94 (18)	C13—C14—C15	109.11 (16)
C2—C3—C6	117.35 (18)	C13—C14—H14A	109.9
C4—C3—C6	123.59 (18)	C15—C14—H14A	109.9
C1—C2—C3	119.7 (2)	C13—C14—H14B	109.9
C1—C2—H2B	120.2	C15—C14—H14B	109.9
C3—C2—H2B	120.2	H14A—C14—H14B	108.3
C13—C9—C7	109.76 (16)	C16—C11—C12	109.60 (17)
C13—C9—H9A	109.7	C16—C11—C8	109.48 (17)
C7—C9—H9A	109.7	C12—C11—C8	108.91 (17)
C13—C9—H9B	109.7	C16—C11—H11A	109.6
C7—C9—H9B	109.7	C12—C11—H11A	109.6
H9A—C9—H9B	108.2	C8—C11—H11A	109.6
			
C7—N2—C6—O1	4.8 (3)	C7—C9—C13—C14	60.4 (2)
C7—N2—C6—C3	−173.33 (16)	C1—N1—C5—C4	−1.6 (3)
C6—N2—C7—C8	174.15 (18)	C3—C4—C5—N1	0.6 (3)
C6—N2—C7—C9	56.1 (2)	N2—C7—C10—C15	−178.66 (16)
C6—N2—C7—C10	−66.6 (2)	C8—C7—C10—C15	−60.6 (2)
C11—C8—C7—N2	−179.08 (15)	C9—C7—C10—C15	58.6 (2)
C11—C8—C7—C9	−60.2 (2)	C11—C16—C15—C10	−60.5 (2)
C11—C8—C7—C10	59.7 (2)	C11—C16—C15—C14	59.4 (2)
C5—C4—C3—C2	0.4 (3)	C7—C10—C15—C16	61.0 (2)
C5—C4—C3—C6	176.27 (18)	C7—C10—C15—C14	−59.6 (2)
O1—C6—C3—C2	23.6 (3)	C5—N1—C1—C2	1.5 (3)
N2—C6—C3—C2	−158.06 (18)	C3—C2—C1—N1	−0.4 (3)
O1—C6—C3—C4	−152.3 (2)	C12—C13—C14—C15	58.6 (2)
N2—C6—C3—C4	26.0 (3)	C9—C13—C14—C15	−61.5 (2)
C4—C3—C2—C1	−0.5 (3)	C16—C15—C14—C13	−58.5 (2)
C6—C3—C2—C1	−176.61 (18)	C10—C15—C14—C13	61.2 (2)
N2—C7—C9—C13	176.98 (16)	C15—C16—C11—C12	−59.6 (2)
C8—C7—C9—C13	60.1 (2)	C15—C16—C11—C8	59.8 (2)
C10—C7—C9—C13	−59.1 (2)	C13—C12—C11—C16	59.6 (2)
C11—C12—C13—C9	60.3 (2)	C13—C12—C11—C8	−60.1 (2)
C11—C12—C13—C14	−59.5 (2)	C7—C8—C11—C16	−59.5 (2)
C7—C9—C13—C12	−60.4 (2)	C7—C8—C11—C12	60.3 (2)

### Hydrogen-bond geometry (Å, º)

**Table:**

*D*—H···*A*	*D*—H	H···*A*	*D*···*A*	*D*—H···*A*
N1—H1*A*···Cl1^i^	0.86	2.19	3.020 (2)	161
N2—H2*A*···Cl1	0.86	2.51	3.272 (2)	148
C1—H1*B*···Cl1^ii^	0.95	2.77	3.520 (3)	136
C2—H2*B*···Cl1^iii^	0.95	2.76	3.494 (3)	134
C5—H5*A*···O1^iv^	0.95	2.31	3.149 (3)	147

Symmetry codes: (i) −*x*+2, −*y*, −*z*+2; (ii) *x*, *y*, *z*+1; (iii) −*x*+1, −*y*, −*z*+2; (iv) *x*+1, *y*, *z*.
